# Comparison of etiology and outcome of dogs undergoing cardiopulmonary resuscitation with different conformation: the Shepherd versus the Bulldog

**DOI:** 10.3389/fvets.2025.1631569

**Published:** 2025-08-29

**Authors:** Junes E. Ossman, Elizabeth A. Rozanski, Ian M. DeStefano, Deirdre Givens Mandryk, Noa Berlin, Sabrina N. Hoehne, Deborah C. Silverstein, Anusha Balakrishnan, for Reassessment Campaign on Veterinary Resuscitation (RECOVER) CPR Registry Consortium

**Affiliations:** ^1^Department of Clinical Sciences, Cummings School of Veterinary Medicine at Tufts University, North Grafton, MA, United States; ^2^University of California at Davis, Davis, CA, United States; ^3^Matthew J Ryan Veterinary Hospital of the University of Pennsylvania, Philadelphia, PA, United States; ^4^Veterinary Emergency Group, Chapel Hill, NC, United States

**Keywords:** cardiopulmonary resuscitation, Reassessment Campaign on Veterinary Resuscitation, defibrillation, mesocephalic, brachycephalic, cardiac arrest, CPR

## Abstract

**Objective:**

To investigate whether chest and head conformation in dogs is associated with the etiology of cardiopulmonary arrest (CPA) and likelihood to experience sustained return of spontaneous circulation (sROSC) following cardiopulmonary resuscitation (CPR).

**Design:**

Retrospective study from the years 2000 and 2023 of dogs that underwent CPR that were one of two body types: either mesocephalic (Shepherd) or brachycephalic (Bulldog).

**Setting:**

Electronic medical records from one veterinary record system and from the Reassessment Campaign on Veterinary Resuscitation (RECOVER) CPR Registry were reviewed.

**Animals:**

A total of 162 dogs were included: 72 in the mesocephalic group (MC) and 90 Bulldogs in the brachycephalic group (BC).

**Measurements and main results:**

Data recorded included signalment, body weight, disease category, whether CPA occurred during general anesthesia, suspected cause of CPA, first identified rhythm on electrocardiogram during CPA, whether defibrillation was performed, whether open chest CPR was performed, whether ROSC was achieved and if it was sustained, and survival to discharge. The BC dogs were more likely to arrest associated with respiratory disease (*p* < 0.001), and MC dogs were more likely to arrest associated with cavity bleeding (*p* = 0.012), trauma (*p* = 0.012), or gastric–dilatation-volvulus (*p* < 0.001). The MC dogs were more frequently defibrillated (*p* = 0.021). Return of spontaneous circulation was achieved in 34.7% (25/72) of MC dogs and 30.0% (27/90) of BC dogs (*p* = 0.522). Survival to discharge was similarly dismal between the two groups (*p* = 0.434) with 2/72 (2.8%) of MC dogs and 1/90 (1.1%) of BC dogs, which reflected both re-arrest and owner decisions. When out-of-hospital CPA cases were excluded, MC dogs were more likely to suffer from a surgical condition (*p* = 0.017) and experienced ventricular fibrillation more often (*p* = 0.032).

**Conclusion:**

Dogs with different head and chest confirmations developed CPA for different reasons, with diseases such as GDV and trauma more commonly affecting MC dogs and respiratory disease more commonly affecting BC dogs. Defibrillation was more common in the MC dogs.

## Introduction

The Reassessment Campaign on Veterinary Resuscitation (RECOVER) guidelines are set to provide evidence-based recommendations for small animal cardiopulmonary resuscitation (CPR) (1, 2). CPR is generally recommended to be performed similarly in both dogs and cats, with the exception of specific guidelines for body conformation in dogs (1–4). The actual clinical relevance of the approach to CPR in different canine body types is unknown, and the cause of cardiopulmonary arrest (CPA) and the effectiveness of CPR in different body conformations are unknown.

Different canine breeds provide a wide spectrum of body conformations, with marked changes in phenotype ranging from tall and keel-chested, such as in German shepherd dogs to wide-chested, brachycephalic conformation such as in English bulldogs (5, 6). It is also important to note that these different breeds can have different facial conformations, with bulldog-type breeds frequently suffering from brachycephalic obstructive airway syndrome (7). All of these breed-specific characteristics could affect the cause of cardiopulmonary arrest (CPA), the arrest rhythm, the necessary intra-arrest interventions, and subsequently the outcome of CPR (8). In addition, brachycephalic dogs may present unique challenges when performing CPR due to their airway anatomy resulting in difficulty intubating for assisted ventilation (5).

Despite advances in veterinary CPR knowledge and education, there is still a paucity of data to evaluate whether physical features such as chest wall conformation and upper airway anatomy alter CPR outcomes. The primary objective of this study was to determine if there are meaningful differences between mesocephalic breeds and brachycephalic breeds regarding their causes of CPA and likelihood to experience sustained return of spontaneous circulation (sROSC). This study chose shepherds to include German Shepherds and Belgian Malinois to represent the mesocephalic breed, more consistent with the original appearance of dogs. sROSC, consistent with RECOVER guidelines, is defined as survival for >20 min following CPR. The hypothesis of this study was brachycephalic breeds would be more likely to experience CPA secondary to respiratory causes and subsequently more likely to have increased ROSC rates as compared to mesocephalic breeds, specifically German Shepherds and Belgian Malinois. We proposed that dogs with brachycephalic conformation would be more likely to have airway obstruction, which could be corrected via prompt intubation and ventilation. A secondary objective was to evaluate whether there were different interventions used during the arrest event between these two populations, specifically defibrillation and open chest CPR.

## Materials and methods

A retrospective study was performed with both the RECOVER CPR Registry (data collected from the years 2016–2021), and electronic medical records from one shared medical record system comprising a university veterinary teaching hospital and one specialty practice associated with the university (data collected from the years between 2000 and 2023) (9, 10). Both databases were used to try to maximize the number of included animals. As our university participates in the RECOVER registry, animals that were identified in both searches were only used once. Inclusion criteria were dogs that underwent recorded CPR and were classified as a Mesocephalic conformation (e.g., German shepherd dog, Belgian Malinois) or Brachycephalic conformation (French or English bulldog). Exclusion criteria were any dog or cat that did not undergo CPR, dogs which did not fall into the breed classifications above, or dogs with an incomplete medical record.

Cases were identified in the electronic medical record by searching for CPR charge codes, which are applied when an unresponsive pet is endotracheally intubated and chest compressions are performed. Data collected included medical record number (to eliminate duplicates), body weight in kilograms, age in years, breed of dog, sex, spay/neuter status, disease category at admission, if general anesthesia was concomitant to the CPA event, suspected cause of event, first identified rhythm on electrocardiogram during CPA, if shockable rhythms (ventricular fibrillation or pulseless ventricular tachycardia) were noted and if defibrillation was performed, if open chest CPR was performed, if recumbency was noted, if ROSC was achieved during CPR, if ROSC was sustained (>20 min) and if the dog was discharged from the hospital. These variables were adapted from the RECOVER CPR Registry, except for disease category at admission and suspected cause of event which were slightly modified as defined below (9, 10). These definitions were applied to all cases regardless of the data source.

The first identified rhythm on electrocardiogram during CPA was documented into four categories: asystole, pulseless electrical activity (PEA), ventricular fibrillation/pulseless ventricular tachycardia (VF/pVT), and unknown/other. The suspected cause of the CPA event was divided into eight categories: traumatic, non-cardiac respiratory (upper airway obstructions and pulmonary parenchymal disease), hemorrhagic cavitary effusions, gastric dilatation and volvulus (GDV)/mesenteric torsion, cardiac, sepsis, neurologic disease, and unknown/other.

### Statistical methods

All statistical analyses were performed using a statistical software package.^1^ Categorical data were compared using a Chi-square test. Continuous data were evaluated for normality with the Shapiro–Wilk test and then compared using either the student’s t-test or the Mann–Whitney U-test as appropriate for distribution. For all comparisons, *p*-values <0.05 were considered significant except for when a Bonferroni adjustment was required. To evaluate for in-hospital arrests only, all comparisons were repeated after removing dogs who presented DOA. When evaluating for overall differences between groups with multiple comparisons, a Bonferroni correction was used to determine an adjusted *p*-value for significance.

## Results

One hundred and sixty-two records (59 from the RECOVER CPR Registry and 113 from the university teaching hospital electronic records system) met the inclusion criteria and were available for review. The cases were classified into two cohorts: mesocephalic dogs (72 dogs comprising 70 German shepherd dogs and 2 Belgian Malinois) and brachycephalic dogs (90 dogs comprising 53 English bulldogs and 37 French bulldogs). Complete demographic data as well as characteristics of the arrest variables are summarized in Table 1.

**Table 1:** Variables associated with all cases of cardiopulmonary arrest and their associated outcomes in mesocephalic and brachycephalic dogs (*n* = 162 dogs).

Variable	Category	Mesocephalic		Brachycephalic		Overall *p*-value	Individual *p*-value
		%	*n*	%	*n*		
Sex	Male intact	20.8	15	28.9	26	**0.004**	0.241
	Male castrated	25.0	18	37.8	34		0.083
	Female intact	5.6	4	12.2	11		0.146
	Female spayed	47.2	34	21.1	19		**<0.001**
	Unknown	1.4	1	0.0	0		0.262
Anesthesia at event	Yes	5.6	4	3.3	3	N/A	0.489
	No	94.4	68	96.7	87		
Suspected cause of event	Traumatic	18.1	13	5.6	5	**<0.001**	**0.012**
	Respiratory (non-cardiac)	9.7	7	48.9	44		**<0.001**
	Hemorrhagic effusion	18.1	13	5.6	5		**0.012**
	GDV/Mesenteric Torsion	22.2	16	1.1	1		**<0.001**
	Cardiac	6.9	5	13.3	12		0.187
	Sepsis	12.5	9	7.8	7		0.317
	Other/unknown	6.9	5	13.3	12		0.187
	Neurologic disease	5.6	4	4.4	4		0.746
First identified rhythm	Asystole	76.4	55	72.2	65	0.123	0.548
	PEA	8.3	6	10.0	9		0.716
	VF	8.3	6	2.2	2		0.074
	Other/unknown	6.9	5	15.6	14		0.091
Defibrillated	Yes	26.4	19	12.2	11	N/A	**0.021**
	No	73.6	53	87.8	79		
Open chest CPR	Yes	13.9	10	8.9	8	N/A	0.314
	No	86.1	62	91.1	82		
ROSC achieved	Yes	34.7	25	30.0	27	N/A	0.522
	No	65.3	47	70.0	63		
Sustained ROSC	Yes	8.3	6	8.9	8	N/A	0.900
	No	91.7	66	91.1	82		

Values in bold represent statistically significant *p*-values. In categories with >2 groups, statistical significance of overall *p*-values was based on Bonferroni correction for multiple comparisons.

The MC dogs had body weights that ranged from 5 kg to 66.2 kg (median 30 kg) and were heavier (*p* < 0.001) than the BC dogs, whose weights ranged from 0.15 kg to 34.5 kg (median 18.8 kg). The average age of the MC dogs was 6.8 (±4.2) years, and the average age of the BC dogs was 5.42 (±4.0). There was no difference in age between the two groups (*p* = 0.05). Of the MC dogs, 15/72 (21%) were intact males, 18/72 (25%) were castrated males, 4/72 (6%) were intact females, 34/72 (47%) were spayed females and 1/72 (1%) had an unknown sex and spay/neuter status. Of the BC dogs, 26/90 (29%) were intact males, 34/90 (38%) were castrated males, 11/90 (12%) were intact females, and 19/90 (21%) were spayed females (*p* = 0.004). The MC dogs were more likely to be spayed females compared to the BC dogs (*p* < 0.001).

There were 4/72 (5.6%) MC dogs and 3/90 (3.3%) BC dogs under general anesthesia at the time of CPA, which was not significantly different (*p* = 0.489). Recumbency was rarely noted in the records and was not further evaluated.

The suspected cause of the event that led to CPA was significantly different between the two groups (*p* < 0.001). The MC dogs were more likely to have died from trauma (*p* = 0.012), hemorrhagic cavitary effusion (*p* = 0.012), or GDV/mesenteric torsion (*p* < 0.001) compared to BC dogs. However, BC dogs were significantly more likely to have suffered CPA from respiratory (non-cardiac) causes (*p* < 0.001) compared to the MC dogs. There was no difference in the suspected cause of CPA between the groups for cardiac causes (*p* = 0.187), sepsis (*p* = 0.317), neurologic disease (*p* = 0.746), or other/unknown causes (*p* = 0.187).

The first identified rhythm was not different between the two groups overall (*p* = 0.123) or when comparing each rhythm individually. The MC dogs were significantly more likely to be defibrillated at any point during CPR (19/72, 26.4%) compared to the BC dogs (11/90, 12.2%, *p* = 0.021). The rhythm that triggered defibrillation attempts was not universally documented. The frequency of open chest CPR performed was 10/72 (13.9%) in MC dogs and 8/90 (8.9%) in BC dogs, which was not significantly different (*p* = 0.314).

There was no difference in the rate of ROSC, with 25/72 (34.72%) of MC dogs and 27/90 (30%) of BC dogs achieving ROSC (*p* = 0.522). sROSC occurred in 6/72 (8.33%) of all MC dogs and 8/90 (8.89%) of all BC dogs, which was also no different (*p* = 0.90). When comparing only those dogs that achieved ROSC in each group, rates of sROSC remained similar, with 6/25 (24.0%) in the MC dogs and 8/27 (29.6%) of BC dogs (*p* = 0.648) achieving sROSC. Out of all dogs that had CPR performed, 2/72 (2.8%) of MC dogs and 1/90 (1.1%) of BC dogs survived to discharge, which was not a statistically significant difference (*p* = 0.434). Non-survival included both re-arrest and owner-elected euthanasia.

Many of the dogs (36/162; 20.9%) presented to the hospital DOA. When the comparisons were repeated to include only those dogs who arrested while in-hospital, the results were similar, with the exception of the suspected cause of arrest and the first identifiable electrocardiogram rhythm during CPA (Table 2). Specifically, the MC dogs that arrested in the hospital were more likely (5/52; 10%) to experience ventricular fibrillation more often than BC dogs (1/74, 1.4%, *p* = 0.032) that arrested in the hospital.

**Table 2:** Variables associated with in-hospital cardiopulmonary arrest and their associated outcomes in mesocephalic and brachycephalic dogs (*n* = 126 dogs).

Variable	Category	Mesocephalic		Brachycephalic		Overall *p*-value	Individual *p*-value
		%	*n*	%	*n*		
Sex	Male intact	17.3	9	31.1	23	**0.005**	0.080
	Male castrated	23.1	12	36.5	27		0.109
	Female intact	5.8	3	9.5	7		0.451
	Female spayed	53.8	28	23.0	17		**<0.001**
	Unknown	0	0	0	0		N/A
Anesthesia at event	Yes	7.7	4	4.1	3	N/A	0.380
	No	92.3	48	95.9	71		
Suspected cause of event	Traumatic	11.5	6	5.4	4	**<0.001**	0.021
	Respiratory (non-cardiac)	13.5	7	50.0	37		**<0.001**
	Hemorrhagic effusion	17.3	9	6.8	5		0.064
	GDV/mesenteric torsion	19.2	10	1.4	1		**<0.001**
	Cardiac	9.6	5	12.2	9		0.654
	Sepsis	15.4	8	9.5	7		0.312
	Other/unknown	5.8	3	9.5	7		0.451
	Neurologic disease	7.7	4	5.4	4		0.604
First identified rhythm	Asystole	78.8	41	67.6	50	0.018	0.164
	PEA	3.8	2	12.2	9		0.104
	VF	9.6	5	1.4	1		**0.032**
	Other/unknown	7.7	4	18.9	14		0.076
Defibrillated	Yes	28.8	15	12.2	9	N/A	**0.019**
	No	71.2	37	87.8	65		
Open chest CPR	Yes	17.3	9	10.8	8	N/A	0.293
	No	82.7	43	89.2	66		
ROSC achieved	Yes	44.2	23	33.8	25	N/A	0.234
	No	55.8	29	66.2	49		
Sustained ROSC	Yes	9.6	5	10.8	8	N/A	0.828
	No	90.4	47	89.2	66		

This is removing dogs that were DOA.

Values in bold represent statistically significant *p*-values. In categories with >2 groups, statistical significance of overall *p*-values was based on Bonferroni correction for multiple comparisons.

Thirty-five dogs underwent CPR before the RECOVER guidelines, and 127 dogs underwent CPR after the RECOVER guidelines were published.

ROSC was documented in 9/35 (26%) pre-RECOVER dogs and 43/127 (34%) post-RECOVER dogs (*p* = 0.41). Sustained ROSC was observed in 3/35 (9%) dogs pre-RECOVER and 11/116 (9%) post-RECOVER dogs (*p* = 1.0).

## Discussion

In this retrospective study, no statistically significant differences were noted between the MC dogs and BC dogs in whether the dogs were under general anesthesia at the time of CPA, first identified cardiac rhythm at the time of CPA, whether open chest CPR was performed, if ROSC was achieved, if ROSC was sustained, or in survival to discharge. Additionally, no difference was seen pre- and post-RECOVER guidelines.

However, MC dogs were more likely to suffer CPA due to trauma, hemorrhagic cavitary effusion, or GDV/mesenteric torsion, while BC dogs were significantly more likely to have developed CPA from non-cardiac respiratory disease. This finding is not unexpected considering the relative frequencies with which these conditions occur in these breeds (11). German shepherd dogs and Belgian Malinois dogs are more likely to succumb to GDV, mesenteric torsion or hemorrhagic effusion (12–14). The finding that BC dogs were more likely to experience CPA secondary to respiratory causes was likely due to their predisposition to brachycephalic obstructive airway syndrome and various gastrointestinal disorders which could lead to sequela such as aspiration pneumonia (7).

Interestingly, the BC dogs that experienced CPA secondary to respiratory disease did not have a higher ROSC compared to the dogs that sustained other causes of CPA. The initial hypothesis of the study was that if cardiopulmonary arrest was in part secondary to airway obstruction prompt intubation could result in improved ROSC. However, as this was not seen, it maybe that brachycephalic mediated airway obstruction did not play a role, or the differences observed reflected the duration of CPA, illness severity, or could simply be due to underpowered results if subtle differences do exist. The duration of CPA was not routinely recorded pre-RECOVER guidelines and was missing in many records. Longer duration of CPA is a poor prognostic sign. Severity of illness scores were also not available for the dogs in this study. A *post hoc* sample size evaluation using the dogs developed CPA while in the hospital, showed that there was only a 21.9% power in identifying a difference, and if there was a 10% difference between groups, 356 dogs per group would be required.

We also found that the use of defibrillation during CPR was significantly higher in MC dogs. Despite the MC dogs experiencing defibrillation more often than the BC dogs, MC dogs did not have an increased rate of initial shockable rhythms reported such as ventricular fibrillation or pulseless ventricular tachycardia (6/72, 8.3%) compared to the BC dogs (2/90, 2.2%, *p* = 0.074). This contradiction could potentially be explained by the fact that the CPR recording sheet prompts the recorder for the initial ECG rhythm during CPA but not necessarily whether shockable rhythms occur throughout CPR. Thus, we suspect that some dogs developed a shockable rhythm that was not recorded on the sheet but subsequently received defibrillation. It is also possible that non-shockable rhythms were treated with defibrillation if the ECG was misinterpreted during the rhythm assessment. Other studies have found that the causes of death for MC dogs more commonly have associated ventricular arrhythmias compared to BC dogs (15, 16). This may be attributed to the small number of dogs with these arrest rhythms. A larger cohort of dogs with shockable rhythms would be helpful to further evaluate this finding.

Prior to the RECOVER guidelines, the ROSC rates in dogs within veterinary centers in the United States ranged from 28 to 60% (17–21). Other studies have previously reported higher ROSC and subsequent discharge rates compared to those in the current study even with similar sample sizes (17, 22). In this study, the low rate of survival to discharge was surprising; a number of dogs were euthanized following ROSC, or CPR was stopped at the owner request. Euthanasia has been reported as the most common cause of death following ROSC in one recent abstract (23). Strong communication prior to undertaking CPA is preferable to owners having to decide under a very stressful moment. Almost all CPR research and studies are affected by the elected euthanasia by owners after initial ROSC.

It has been reported that dogs requiring in-hospital CPR have higher success rates for achieving ROSC, sustaining ROSC and subsequently surviving to discharge compared to dogs who experience CPA outside of the hospital (3). This seems intuitive that pets who experience CPA in the hospital receive CPR faster and therefore have improved outcomes (1, 3, 4). When evaluating the data for in-hospital incidence of CPA only, it showed very similar data comparisons but exposed that MC dogs more often present with a surgical condition (e.g., GDV) and experienced ventricular fibrillation compared to the BC dogs. This re-affirms the suspicion that many of the MC dogs experience surgical disease processes which often are associated with ventricular arrhythmias (15, 16).

This study has numerous important limitations to consider. The CPR record and medical records were sparse. Cases may not have been captured if the CPR charge code was not entered. We also cannot comment or analyze whether the position of recumbency of the dog during CPR was associated with outcome, as this was not routinely recorded. In addition, the suspected cause of the CPA event in some cases could have been subjective or not definitively determined, which could have biased the data. In some cases, CPR may have been discontinued per the owner’s request when ROSC otherwise may have been achieved. The retrospective nature of the study removes any standardization of treatment or care. Additionally, the study covered a large period of time both before and after the RECOVER guidelines were published, which could have skewed the outcomes although no difference was seen in comparing the groups. Also, the presence and severity of brachycephalic obstructive airway syndrome in the BC dogs and whether they had previously undergone corrective surgery were not documented, which may have influenced the results of the BC group. Finally, as shepherds and Belgian Malinois were used as mesocephalic dogs, they may not be representative of all non-brachycephalic dogs. Different results might have been seen with other breeds.

This is the first research study to investigate breed-related outcomes for CPR. Despite brachycephalic related airway obstruction, no differences in outcome were observed. On-going evaluation of specific categories of CPA may provide useful.

## Data Availability

The raw data supporting the conclusions of this article will be made available by the authors without undue reservation.

## Footnotes

^1^SPSS Statistics, IBM, Armonk, NY.
